# LoRa Communications Spectrum Sensing Based on Artificial Intelligence: IoT Sensing

**DOI:** 10.3390/s25092748

**Published:** 2025-04-26

**Authors:** Partemie-Marian Mutescu, Valentin Popa, Alexandru Lavric

**Affiliations:** Faculty of Electrical Engineering and Computer Science, Ștefan Cel Mare University of Suceava, 720229 Suceava, Romania; marian.mutescu@usm.ro (P.-M.M.);

**Keywords:** artificial intelligence, internet of things, LoRa, LPWAN, spectrum sensing

## Abstract

The backbone of the Internet of Things ecosystem relies heavily on wireless sensor networks and low-power wide area network technologies, such as LoRa modulation, to provide the long-range, energy-efficient communications essential for applications as diverse as smart homes, healthcare, agriculture, smart grids, and transportation. With the number of IoT devices expected to reach approximately 41 billion by 2034, managing radio spectrum resources becomes a critical issue. However, as these devices are deployed at an increasing rate, the limited spectral resources will result in increased interference, packet collisions, and degraded quality of service. Current methods for increasing network capacity have limitations and require advanced solutions. This paper proposes a novel hybrid spectrum sensing framework that combines traditional signal processing and artificial intelligence techniques specifically designed for LoRa spreading factor detection and communication channel analytics. Our proposed framework processes wideband signals directly from IQ samples to identify and classify multiple concurrent LoRa transmissions. The results show that the framework is highly effective, achieving a detection accuracy of 96.2%, a precision of 99.16%, and a recall of 95.4%. The proposed framework’s flexible architecture separates the AI processing pipeline from the channel analytics pipeline, ensuring adaptability to various communication protocols beyond LoRa.

## 1. Introduction

The Internet of Things (IoT) transformed many industries and daily life by connecting the physical world to the Internet through devices that can collect, interact, and send data to the Internet using sensors, actuators, and wireless communication protocols [1]. With this in mind, wireless sensor networks (WSNs) have become the key enablers of the IoT ecosystem, using low-power wide area network (LPWAN) technologies to send and receive data from gateways and sink nodes [2]. LPWAN technologies offer long-range and energy-efficient communication that makes them suitable for many IoT applications, such as smart homes [3], healthcare [4], agriculture [5], smart grids [6], and transportation [7]. The IoT market experienced significant growth due to the versatility of its applications. Research shows that the number of IoT devices is growing by approximately 2 billion each year. Projections show that there will be around 41 billion connected IoT devices by the year 2034 [8]. Most LPWAN technologies use unlicensed frequency bands, such as the 868 MHz short-range devices (SRD) band, the 915 MHz band, and the 2.4 GHz industrial, scientific, and medical (ISM) band [9,10,11]. Each of these bands has its own bandwidth limitations. For example, the 868 MHz SRD band has only 10 MHz of available bandwidth, while the 915 MHz and 2.4 GHz bands have 28 MHz and 100 MHz of available bandwidth, respectively. Currently, one of the most widely adopted LPWAN communication technologies worldwide is LoRa [12], which accounts for approximately 40% of global IoT connectivity. The increasing use of LoRa in IoT networks with limited radio resources presents several challenges, including higher packet collision rates, interference, and reduced capacity, which can lead to lower quality of service (QoS). Current methods of increasing scalability, such as duty cycle regulations [13] and redundancy mechanisms [14], are not always effective in addressing these issues, especially in large-scale, high-density deployments. Recent research proposed alternative modulation techniques aimed at improving performance in LPWAN environments. One example is dual-mode chirp spread spectrum (DM-CSS) modulation [15], which enhances spectral efficiency by simultaneously multiplexing even and odd chirp signals with phase shifts, resulting in a significant increase in the number of transmitted bits per symbol. DM-CSS demonstrated higher spectral and energy efficiency compared to classical LoRa modulation, making it a promising candidate for future IoT networks operating in crowded unlicensed spectrum environments.

Another approach is represented by advanced solutions, such as spectrum sensing (SS) techniques. These techniques allow us to detect and identify different wireless protocols operating within shared frequency bands [16,17,18,19]. This allows the IoT network operators to dynamically allocate frequencies and reduce interference. This approach is critical to maintaining optimal performance, reliability, and scalability of LoRa-based IoT networks, ensuring their continued growth and integration into future IoT infrastructure.

In addition, advanced channel analytics provide network operators with insights to evaluate scalability issues within the LoRa network architecture. Optimal allocation of spreading factors (SF) is essential to mitigate the capture effect and enhance network capacity [20]. In large-scale, high-density LoRa networks, the capture effect occurs at the gateway when multiple LoRa transmissions simultaneously arrive on the same communication channel and use identical SFs. Under these conditions, the gateway successfully demodulates only the signal with the highest received power, while all other transmissions are discarded, increasing packet error rates. Nevertheless, due to LoRa’s use of SFs that are theoretically orthogonal [21], a gateway can typically extract and demodulate multiple signals transmitted simultaneously on the same frequency channel, provided that different SFs are used. This orthogonality is a fundamental property that underpins the scalability of LoRa-based networks, allowing multiple devices to share limited spectrum resources efficiently. However, it is important to recognize that perfect orthogonality is difficult to achieve in practical applications. Real-world factors such as timing misalignments between transmitters, carrier frequency offsets, and differences in received signal power can introduce imperfections that reduce the degree of orthogonality. These impairments may lead to inter-SF interference, especially in dense network scenarios where simultaneous transmissions are more frequent. Several studies, such as [22], analyzed the impact, demonstrating that LoRa’s performance under real-world conditions can deviate from the idealized assumptions often made in theoretical models. In this context, while LoRa SFs offer strong resilience to interference under many operating conditions, it is important for network designers and operators to consider potential cross-interference when planning large-scale IoT deployments. Proper SF allocation strategies, along with synchronization and power control mechanisms, can help mitigate the practical limitations of SF orthogonality and improve overall network performance.

Live network monitoring tools based on SS techniques can help operators track how SF is being allocated. These tools can identify edge cases where sensors excessively use either SF7, which is good for low-latency applications, or SF12, which supports long-range but uses more energy, has higher time-on-air (ToA), and higher collision risks. The SS techniques make LoRa networks more adaptable by allowing SF assignments based on the sensing results. This helps address network optimization from a latency, power, and range perspective according to the specific needs of IoT applications. It also provides deeper insight into network behavior, enabling proactive intervention and more accurate troubleshooting of devices that consistently use inefficient SFs schemes.

The importance of spectral constraints and SF allocation in LoRa networks has been studied previously. Lavic et al. [23] have shown that a maximum number of 8000 nodes can simultaneously transmit LoRa packets to a gateway with an imposed duty cycle of 1%. Farhad et al. [24] propose a DL-based LoRa ADR mechanism that increases the packet success rate by up to 15% with 600 transmitting LoRa nodes. Housem et al. [25] propose and evaluate an artificial intelligence (AI)-based LoRaWAN optimization method using auto-regressive algorithms, support vector machines (SVMs), and temporal fusion transformers (TFTs). The authors employ these algorithms for network behavior prediction. Based on these predictions, they propose an optimization mechanism that conserves a percentage of the available duty cycle for anticipated increase in LoRaWAN network traffic.

Aohan et al. [26] proposes and evaluates an RL-based resource allocation mechanism for LoRaWAN networks. In his approach, the learning agent is represented by a LoRaWAN node that can choose its communication channel and SF, transmits the data frame to the LoRaWAN gateway, and receives an ACK or NACK response. If the transmission receives an ACK, the LoRaWAN node is rewarded, otherwise it is penalized. According to Aohan et al. [26], the proposed methodology can maintain an FSR of 1 for up to six simultaneously transmitting LoRaWAN devices, with the FSR dropping to 0.5 when the number of devices increases to eight. This method is compared to the random channel and SF selection mechanism, which has an FSR of 0.4 at six LoRa nodes, dropping below 0.3 when increasing to eight LoRaWAN nodes.

The LoRaWAN network enhancement methods presented so far are based on metrics that can be obtained directly from the MAC layer via radio packet metadata. However, in order to obtain MAC layer metrics for these methods, the LoRa packets must first be received and demodulated. In addition, these methods do not take into account other communication protocols that may be operating in the same unlicensed frequency bands. With this in mind, network sensing must be performed at the physical link layer using SS techniques.

Shahid et al. [27] analyze the performance of convolutional neural networks (CNNs) for detecting and classifying LPWAN radio communication protocols, including Sigfox, LoRa, and IEEE 802.15.4g [28] based on IQ samples input. Their study achieved a classification accuracy of 95% at SNR levels above 10 dB. However, classification accuracy significantly decreases at lower SNR values, dropping below 70% at 0 dB and further declining to approximately 25% at −10 dB. Almohamad et al. [29] propose an enhancement to the methodology described in [27], focusing specifically on classifying LoRa radio transmissions according to their SFs, using cyclostationary features as input data for a CNN model. According to their results, the cyclostationary features signal representation achieves a classification accuracy of approximately 99% at an SNR of −10 dB.

In our previous work [30], we designed and evaluated DL-based spectrum sensing techniques for LoRa SF detection using spectrograms as the input. Using a proprietary dataset consisting of spectrograms of LoRa radio transmissions in the −20 to 30 dB SNR range, we trained and evaluated an image classification CNN for LoRa SF detection, obtaining a classification accuracy of 98%. However, this methodology can be applied when the spectrogram image contains only one LoRa transmission. In [31], we extend the capabilities of the approach in [30] to the simultaneous detection of multiple LoRa and Sigfox radio transmissions within a spectrogram. The developed DL spectrum sensing network was based on the second iteration of You Only Look Once (YOLO) [32].

Compared to our previous work [30,31], which primarily focused on deep learning-based LoRa SF detection using spectrogram inputs, in this paper, we propose and evaluate a hybrid SS framework specifically designed for SF detection in LoRa modulation and advanced channel analysis. Our proposed framework combines traditional signal processing techniques and advanced AI models to detect, classify, and extract detailed channel information such as center frequency, bandwidth, and occupancy level for multiple simultaneous LoRa transmissions within a wideband signal. It relies entirely on IQ sample processing to ensure accurate and efficient signal analysis. A key feature of our approach is the separation of the AI processing pipeline from the advanced analysis pipeline, making the framework more flexible.

This paper makes several important contributions: it develops a new hybrid framework that combines signal processing and AI for effective LoRa SF detection and classification, introduces robust methods for advanced channel analysis directly from IQ samples, designs a flexible architecture by separating the processing pipelines to support different communication standards, and presents a thorough evaluation of the effectiveness, and reliability of the framework in realistic scenarios.

The remainder of this paper is organized as follows: Section 2 provides an overview of the proposed SS framework. Section 3 details the transmission detection and clustering algorithm, which is specifically designed to detect and isolate individual radio transmissions within a wideband signal and prepare them for subsequent analysis. Section 4 outlines the methodology used to create the dataset used in the training and evaluation of the LoRa SF detection AI model. Section 5 describes the design, training procedures, and evaluation of this LoRa SF detection model. Section 6 provides a description of the channel analytics processing pipeline and a comprehensive evaluation of the overall framework through two live deployment scenarios involving software-defined radio (SDR) devices and dedicated LoRa transceivers. Finally, Section 7 summarizes the key findings and concludes the paper.

## 2. Spectrum Sensing Framework for LoRa Modulation Detection and Communication Channel Analytics

In this section, we present the system design of our proposed SS framework for LoRa modulation detection and communication channel analytics. We consider a wideband signal $x\left(n\right)$ subjected to typical wireless channel impairments, including Rayleigh fading and additive white Gaussian noise (AWGN), potentially containing multiple concurrent LoRa transmissions. The principal objective of this framework is the accurate detection and classification of each LoRa transmission within the received wideband signal, along with detailed channel analytics for every identified transmission.

**Figure 1:**
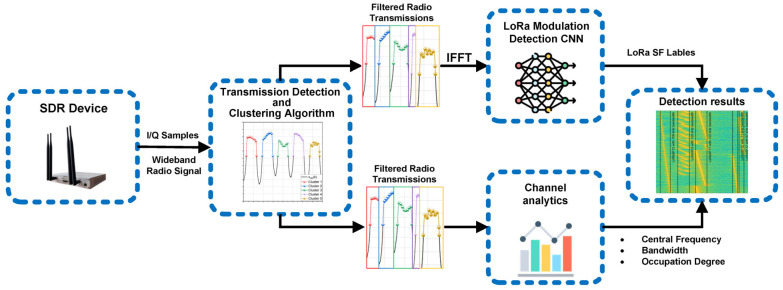
Spectrum sensing framework for LoRa modulation detection and communication channel analytics.

In Figure 1, we provide a graphical representation of the framework. This hybrid SS approach integrates classical detection techniques, such as energy detection and signal processing, with AI-driven solutions based on CNNs. By combining these complementary methods, we enhance both the accuracy and robustness of LoRa detection and classification.

The SS algorithm Input comprises IQ sample streams produced by an SDR device, at the baseband frequency, that captures the wideband radio signal in the target frequency band. These IQ samples represent the real and imaginary components of the wideband signal, enabling the extraction of time domain features. The power of the received signal is normalized using (1) and (2), where $P_{t}$ denotes the power of the wideband signal $x\left(n\right)$, N is the number of IQ samples of $x\left(n\right)$, and $x_{n}\left(n\right)$ is the resulting normalized signal. The normalization process reduces the computational overhead, enhances the processing capabilities of the CNN both during training and inference, and improves the model convergence.

(1) $$P_{t}=\frac{1}{N}{\sum_{n=1}^{N}\left|x(n)\right|}^{2}.$$

(2) $$x_{n}(n)=\frac{x(n)}{\sqrt{P_{t}}}.$$

Following normalization, the signal is passed through a transmission detection and clustering algorithm, which will be presented in the following section, that identifies and isolates individual radio transmissions within the wideband signal. Each identified transmission is then passed to two separate processing pipelines. The first pipeline is represented by the AI model for LoRa modulation detection. This processing pipeline identifies the LoRa SF using a time series CNN taking IQ samples as input. The second processing pipeline is represented by the communication channel analytics algorithm. The two processing pipelines can be individually used, making the SS framework more versatile. Finally, the SS algorithm outputs both the detection results and the corresponding channel analytics.

## 3. Transmission Detection and Clustering Algorithm

This section presents the design and implementation of the transmission detection and clustering algorithm (TDCA), which enables the SS framework to identify and separate individual radio transmissions within the received wideband signal and prepare them for further processing trough the two processing pipelines.

As presented in the introduction of this section, the SS framework input receives a wideband signal at the baseband frequency that can contain multiple radio transmissions. However, considering that a CNN can predict a single modulation class for the input signal, the individual radio transmissions within the wideband signal must be firstly separated in frequency and filtered individually.

For this, we implemented a transmission detection and clustering algorithm based on traditional SS approaches [33] and signal processing. Firstly, the TDCA uses the normalized $x_{n}\left(n\right)$ wideband signal, which is converted to the frequency domain by applying a discrete Fourier transform using (3), where N is the number of IQ samples in $x_{n}\left(n\right)$, and $x\left(k\right)$ is the representation of $x_{n}\left(n\right)$ in the frequency domain.

(3) $$x(k)=\sum_{n=0}^{N-1}x_{n}(n)\;e^{-j\frac{2\pi kn}{N}}.$$

After obtaining the frequency domain representation of the wideband signal, the 0 Hz component is shifted to the center of the spectrum, using (4). In this operation, the frequency associated with each discrete index k is mapped to a real frequency according to (5), where $F_{s}$ is the sampling frequency and N is the number of samples. After shifting, the frequency axis spans from $-F_{s}/2$ to $F_{s}/2$, aligning the discrete bins with their corresponding physical frequencies. The magnitude is then represented as power spectral density (PSD) using (6).

(4) $$x_{shifted}(k)=\left\{\begin{array}{c} x(k+N/2),0\leq k<N/2\\ x(k-N/2),N/2\leq k<N \end{array}\right..$$

(5) $$f(k)=\left(\frac{k}{N}-\frac{1}{2}\right)F_{s}.$$

(6) $$x_{dB}(k)=20\log_{10}\left(\frac{\left|x_{shifted}(k)\right|}{N}\right).$$

A noise suppression is performed using a moving average operation, as described in (7) and (8), where (7) defines the window size *w* as the floor of 1% of N and N represents the number of frequency bins in $x_{db}\left(k\right)$. Equation (8) then updates the $x_{db}\left(k\right)$ values by applying the moving average operation.

(7) $$w=\left\lfloor \frac{N}{100}\right\rfloor .$$

(8) $${x_{dB}}^{\prime }(k)=\frac{1}{w}\sum_{m=\max(1,k-\frac{w}{2})}^{\min(N,k+\frac{w}{2})}x_{dB}(m).$$

Each frequency bin in the ${x_{db}}^{\prime }\left(k\right)$ is compared to a threshold determined by the mean value of ${x_{db}}^{\prime }\left(k\right)$. Any frequency bin index that exceeds this threshold is stored in a vector. This approach is similar to the energy detection [33] method commonly used in spectrum sensing algorithms for identifying occupied and unoccupied frequency bands. However, in this work, this method is used specifically to identify frequency bins that may be part of clusters representing a distinct narrowband radio transmission in the $x\left(n\right)$ wideband signal.

After determining the relevant frequency bin indexes, the difference between adjacent indexes is calculated and compared against a predefined threshold. This comparison helps determine whether the adjacent indexes belong to the same cluster or to separate clusters. Through multiple tests and empirical studies, an index difference of 0.3% of the total number of frequency bins was found to generate optimal clustering results.

**Figure 2:**
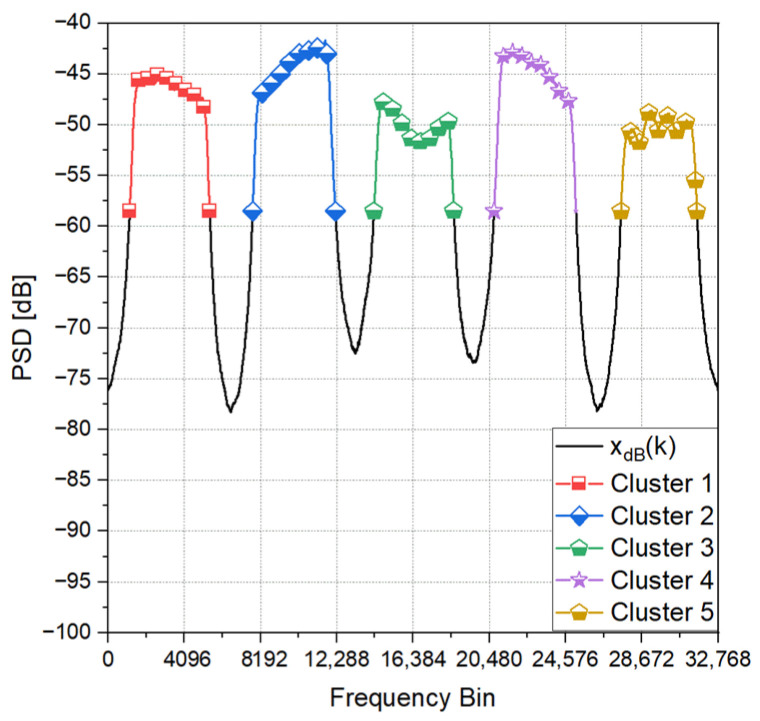
Transmission detection and clustering algorithm applied on a 1 MHz radio signal containing five LoRa transmissions.

Once preliminary clusters are formed, an additional filtering step is applied. Clusters that exceed the imposed PSD threshold but do not contain a sufficient number of frequency bins to represent a radio transmission are discarded. Figure 2 presents an example of the TDCA applied on a wideband signal containing five distinct LoRa transmissions.

After the clusters representing individual radio transmissions are identified, their frequency boundaries are mapped onto the overall wideband frequency range, extending from $-F_{s}/2$ to $F_{s}/2$, where $F_{s}$ is the sampling frequency of $x\left(n\right)$. A passband filter with a transition band of 10 kHz is then designed for each cluster according to its frequency boundaries and applied in the frequency domain to suppress all other signal components. The resulting signals, each containing only one cluster, are transformed back into the time domain via an inverse discrete Fourier transform, as indicated in (9), where $x_{f}\left(k\right)$ is the filtered signal represented in the frequency domain, N is the number of frequency bins in $x_{f}\left(k\right)$, and $x_{f}\left(n\right)$, is the resulting filtered signal in the time domain. Finally, each single-transmission signal is shifted to the center of the baseband using (10). This is accomplished by multiplying the filtered signal $x_{f}\left(n\right)$ by a complex exponential of unit amplitude at a frequency equal to the negative of the cluster’s center frequency. In (10), $f_{c}$ is the central frequency of the signal cluster, n denotes the discrete time sample index and spans from 0 to N−1. The center frequency $f_{c}$ is a real-valued offset within [$-F_{s}/2$ $F_{s}/2$].

(9) $$x_{f}(n)=\frac{1}{N}\sum_{k=0}^{N-1}x_{f}(k)\;e^{j\frac{2\pi }{N}kn}.$$

(10) $${x^{\prime }}_{f}(n)=x_{f}(n)\;e^{-j2\pi \frac{f_{c}}{F_{s}}n}.$$

At the end of this process, the TDCA produces multiple single-transmission signals corresponding to the number of detected signal clusters. These signals are then passed to the CNN processing pipeline for LoRa modulation detection, and to the channel analytics processing pipeline for central frequency, bandwidth, and occupation degree computing. Although the current implementation is optimized for LoRa signals, the TDCA is based on general spectrum sensing principles and can be adapted to alternative narrowband modulations such as FSK or NB-IoT. Adapting the framework would involve tuning the frequency resolution, adjusting thresholding criteria based on the new signal characteristics, modifying the passband filter parameters to match different bandwidths, and retraining the CNN classifier using features representative of the target modulation scheme. The modular structure of the framework facilitates such adaptations with minimal architectural changes.

## 4. Dataset Creation

The first step in designing, training, and evaluating the LoRa modulation detection CNN is creating a comprehensive dataset. This dataset encompasses six LoRa spreading factors (SF7 through SF12), each corresponding to the number of bits that can be encoded in a LoRa symbol. Using MATLAB 2023a and the library developed by Al Homsi et al. [34], we generated 26,000 signal samples for each spreading factor at a sampling rate of 1 MS/s. Each sample employed random byte sequences as the LoRa packet payload, a transmission power of 14 dB following European LoRa Regional parameters [35], and a bandwidth of 125 kHz.

Each signal sample was then passed through a channel model simulating various propagation impairments, including Rician multipath fading (for urban, suburban, and rural environments), Doppler shift, clock offset between the transmitter and receiver, and different SNR conditions imposed via additive white Gaussian noise (AWGN).

Because LoRa can operate below the noise floor, we considered 26 SNR values ranging from −20 dB to 30 dB with an increment of 2 dB, generating 1000 samples at each SNR level. The multipath fading model used path gains of 0 dB, −2 dB, and −10 dB, and the maximum Doppler shift was set to 4 Hz at a carrier frequency of 868 MHz. Additionally, the clock offset was randomly varied between −5 ppm and 5 ppm for both the carrier frequency and the sampling rate.

**Figure 3:**
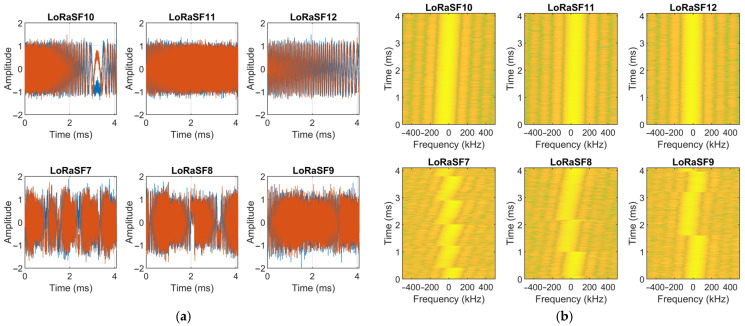
Samples of the generated dataset used for training and evaluating the LoRa SF detection CNN. (**a**) Amplitude/time plots (Blue—In Phase, Red—Quadrature); (**b**) spectrogram plots.

Each LoRa signal sample was truncated to 4096 consecutive IQ samples, which is the minimum amount of IQ samples required to capture an SF9 symbol at a 1 MS/s sampling rate, and provides a sufficient amount of samples to differentiate between SF10, SF11, and SF12 symbols. Since every LoRa transmission starts with eight consecutive upchirps, the 4096 samples were selected starting with a random offset to enhance dataset diversity with respect to symbol distribution. Figure 3 illustrates examples of the resulting dataset through time/amplitude (a) plots and spectrograms (b). In the time/amplitude plots, the I and Q channels are represented in blue and red, respectively, while the spectrogram images are plotted with a window size of 256. The complete dataset, containing 156,000 signal samples, is publicly available on GitHub [36].

## 5. Design, Training, and Evaluation of the LoRa Modulation Detection Convolutional Neural Network

**Figure 4:**
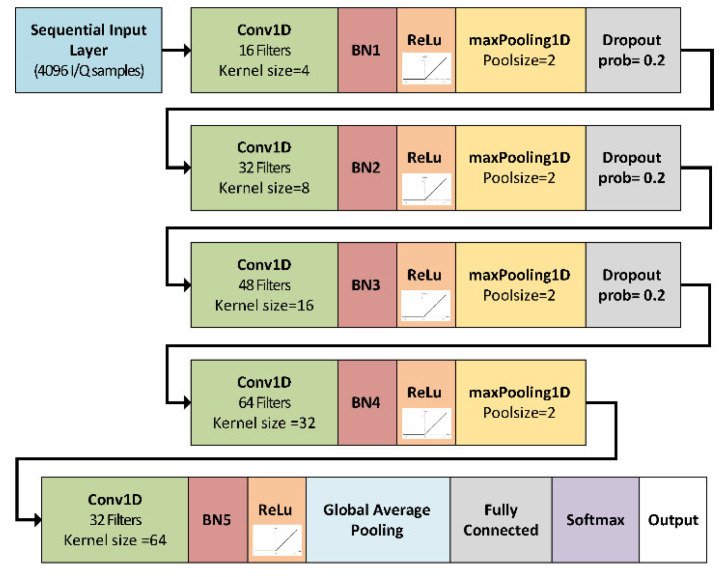
Designed LoRa SF detection convolutional neural network structure.

The proposed CNN architecture adopts a classical hierarchical structure, as shown in Figure 4. It begins with a sequence input layer configured to receive a minimum of 4096 samples, which is sufficient to capture a LoRa SF9 symbol at a 1 MS/s sampling rate, but can also receive signal frames with more than 4096 samples. Within this layer, z-score normalization is applied, and the complex input samples are split into real and imaginary components.

After the input layer, the network is organized into multiple hierarchical blocks. In the first block, there is a 1D convolutional layer (Conv1D) with a filter size of 4 and 16 filters. This block also includes a batch normalization layer, a ReLU activation layer, a max pooling layer, and a dropout layer. In subsequent blocks, the filter size doubles incrementally to 8, respectively, 16, and the number of filters increases by 16 in each block. Max pooling and dropout layers help mitigate overfitting.

In the fourth block, the Conv1D layer continues to increase filter size to 32 and filter count to 64, but the dropout layer is removed. In the fifth block, the Conv1D layer has a filter size of 64 and 32 filters, and the max pooling layer is replaced by a global average pooling layer. The global average pooling layer aggregates the extracted features into a single vector, which is then passed to a fully connected layer and a softmax layer to produce class probabilities for the different LoRa SF categories.

The selected filter sizes are derived from the number of IQ samples (1024) required to represent a LoRa SF7 symbol at a 1 MS/s sampling rate. By using smaller filter sizes in the initial layers, the CNN can capture localized features such as transient events or short-term patterns. In deeper layers, larger filter sizes enable the extraction of broader contextual information. The resulting network has a total of 26 layers with 258,950 trainable parameters.

**Figure 5:**
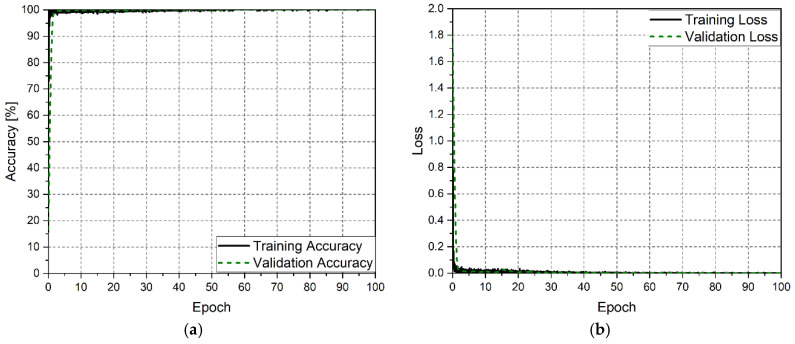
Performance parameters for the training process for the LoRa convolutional neural network algorithm. (**a**) Training and validation accuracy; (**b**) training and validation loss.

For the training and evaluation of the CNN, the dataset was partitioned into 70% for training, 15% for validation during the training process, and 15% for testing. The CNN was trained over a total of 100 epochs. Considering that the network was initialized without pretraining, an initial learning rate of 0.3 was set, with a drop factor of 0.75 applied every six epochs. Training was conducted using the stochastic gradient descent with momentum (SGDM) optimizer. A batch size of 256 was employed, and validation was performed at the end of each training epoch. Additionally, to mitigate overfitting and enhance the CNN’s generalization capabilities, the training dataset was shuffled at the beginning of each epoch. The training hardware platform included an Alienware Aurora R10 system (AMD Ryzen9 3950X, 64GB DDR4, and 2xGeForce RTX 3060 6GB). The training process was evaluated using accuracy and loss metrics, the progression of which is presented in Figure 5a and Figure 5b, respectively. The CNN converged within the initial training epochs, achieving 98% accuracy after only four epochs.

Following the training process, the CNN was evaluated under two distinct testing scenarios. In the first scenario (scenario A), it was tested on a partition of the original empirically generated dataset with emulated channel impairments, comprising 3900 signal samples per LoRa SF. In this scenario, the CNN achieved 100% accuracy. To eliminate the possibility of overfitting, a second testing dataset was generated. This secondary dataset contained 2600 samples for each LoRa SF, with 100 signal samples for each SNR value. On this dataset, the CNN achieved an accuracy of 99.69%.

**Figure 6:**
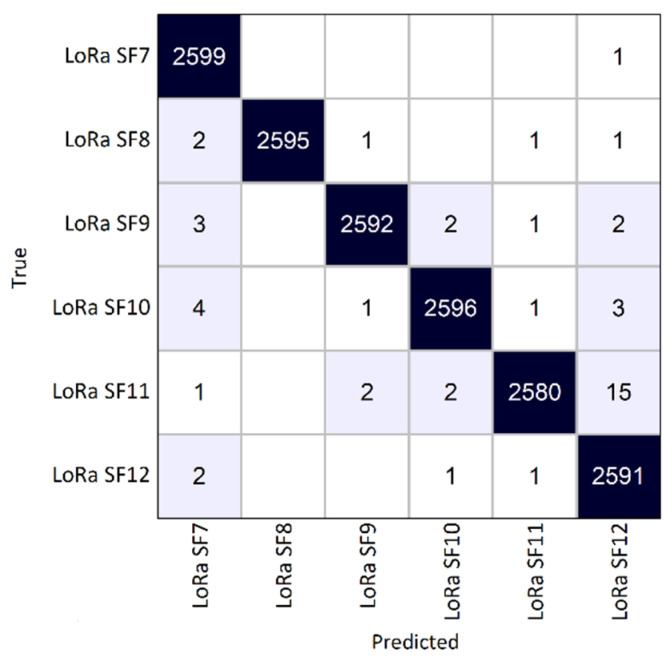
Confusion matrix for empirical data testing (test scenario A).

From the confusion matrix shown in Figure 6, it can be seen that classification consistency among the LoRa SF classes is high, with the majority of signal samples correctly classified. This shows that the CNN successfully captured the features of different SFs and is capable of generalizing. Most misclassifications occurred between LoRa SF11 and LoRa SF12, and in every SF from 8 to 12, there was at least one misclassification as SF7. Nonetheless, the number of misclassified signals is minimal compared to the total number of analyzed LoRa signal samples.

In the second test scenario (scenario B), we conducted real-world testing using SDR devices positioned indoors at a distance of 10 m. An Adalm Pluto SDR [37] was configured on the transmitter side to continuously transmit 5000 LoRa packets for each spreading factor (SF). On the receiver side, an Ettus USRP N310 SDR [38] was set up to receive the LoRa signal samples in IQ format and pass them on to the CNN for classification. Both SDRs were set to a center frequency of 868.1 MHz with a sampling rate of 1 MS/s.

**Figure 7:**
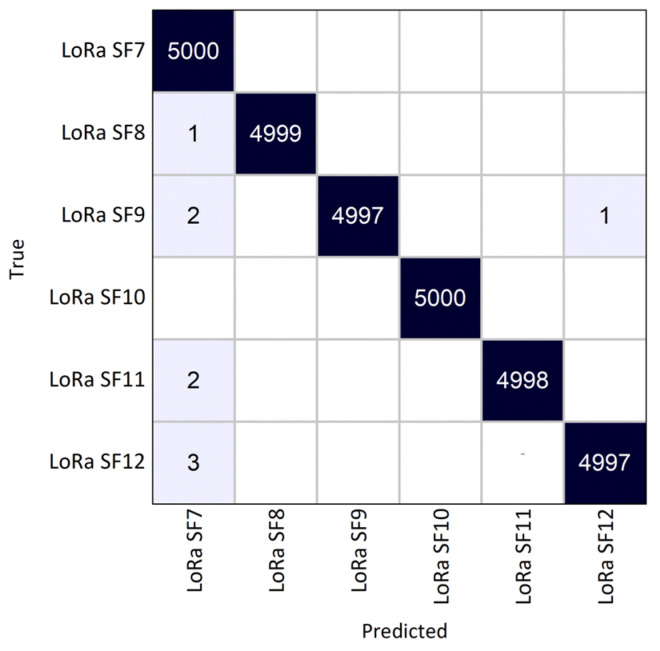
Confusion matrix for a real operating environment (test scenario B).

Under these conditions, the CNN achieved an accuracy of 99.97%. From the confusion matrix in Figure 7, we can see that each SF is correctly identified, even in a real-world testing scenario. Although the SF8-to-SF12 misclassification with SF7 observed earlier is also present here, the CNN maintains a high level of performance in this evaluation scenario.

## 6. Radio Channel Analytics and Live Testing

In this section, we present the radio channel analytics processing pipeline along with the live testing and implementation of the proposed SS framework. As presented in Section 2, the second processing pipeline of the SS framework involves computing channel analytics for both the overall frequency band and each detected transmission. For each identified radio transmission, the SS framework reports the central frequency, bandwidth, and SF if the radio transmission uses LoRa modulation. The central frequency is determined during preprocessing of the wideband signal using the TDCA. Since each transmission is isolated by a passband filter with a 10 kHz transition band, the bandwidth is calculated using the 99% occupied bandwidth method. This approach identifies the lower ($f_{lo}$) and upper ($f_{hi}$) frequency boundaries that account for 99% of the total signal power, satisfying (11), where $P_{total}$ is the total power of the signal from $-F_{s}/2$ to $F_{s}/2$, and $P\left(f\right)$ is the power of the signal at frequency f.

(11) $$\int_{f_{lo}}^{f_{hi}}P(f)df=0.99P_{total}.$$

Beyond the individual channel analytics for the detected LoRa transmissions, the overall occupancy degree of the analyzed frequency band is computed using (11). In this expression, M is the total number of detected transmissions, $T_{BW}$ is the total bandwidth of the analyzed frequency band, and $BW\left(m\right)$ is a vector containing the bandwidth values of each detected LoRa transmission.

(12) $$OD=\frac{100}{T_{BW}}\sum_{m=1}^{M}BW(m).$$

**Figure 8:**
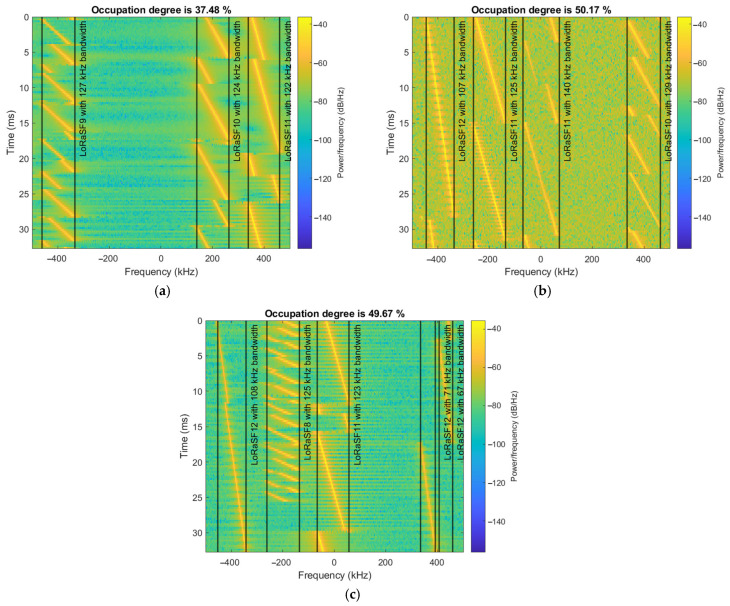
Spectrum sensing framework for LoRa modulation detection and communication channel analytics output examples. (**a**) Channel occupancy degree of 37.48%; (**b**) channel occupancy degree of 50.17%; and (**c**) channel occupancy degree of 49.67%.

In Figure 8, three examples of the SS framework outputs are shown. The detected LoRa transmissions are bounded by the determined frequency limits. By measuring the symbol time for each detection, it can also be observed that the CNN correctly identified the LoRa spreading factors. In Figure 8c, due to the long symbol time of the LoRa SF12 transmission, it was identified as two separate LoRa SF12 transmissions with smaller bandwidths. Nonetheless, the overall occupancy degree of the analyzed frequency band remains accurate.

The next step is to perform the live implementation and evaluation of the SS framework for LoRa SF detection. As described in the previous section, the developed CNN was trained and evaluated on a dataset of 156,000 empirically generated LoRa signal samples, which included simulated channel impairments with SNR values ranging from −20 dB to 30 dB. This resulted in a classification accuracy of 99.69%. A second evaluation scenario involved two SDR devices deployed in an indoor environment at a distance of 10 m, resulting in an accuracy of 99.97%. However, to fully assess the performance of the SS framework, a third evaluation scenario was conducted. In contrast to the previous test setups, this scenario uses IoT nodes equipped with LoRa radio transceivers.

**Figure 9:**
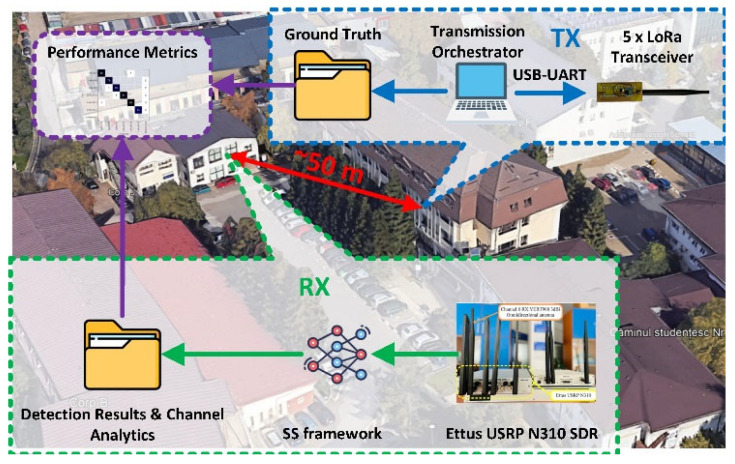
Spectrum sensing framework live implementation. Test setup for real-world evaluation using dedicated LoRa transceivers.

From Figure 9, which illustrates the test setup (scenario C) for evaluating the SS framework in a real-world environment, we see that the setup consists of two main components:

- TX (transmitter): Five IoT nodes using LoRa transceivers, connected to a transmission orchestrator.
- RX (receiver): A host machine running the SS framework along with an SDR device for capturing signals.

The transmitter and receiver were placed approximately 50 m apart, in separate buildings, indoors. In this setup, we evaluate the performance of the SS framework using several metrics that assess both the TDCA and the CNN:

- Detection accuracy: Represents the total number of detections made by the TDCA relative to the total number of LoRa transmissions. This metric reflects the TDCA algorithm’s effectiveness in detecting and separating individual narrowband transmissions within the wideband signal, and it indicates the SS framework’s capability to correctly report the occupancy status of a communication channel.
- Precision: the proportion of correctly classified LoRa transmissions among all detected LoRa transmissions.
- Recall: the proportion of correctly detected and classified LoRa transmissions out of the total transmissions sent by the IoT nodes and transmission orchestrator.
- Confusion matrix: shows instances of misclassification among the detected LoRa spreading factors.
- Mean absolute central frequency deviation (MACFD): measures the average deviation of the detected LoRa transmissions central frequencies from the known central frequencies.
- Mean absolute bandwidth deviation (MABD): measures the average deviation of the calculated bandwidths for the detected LoRa transmissions from their known bandwidth.

For these metrics, a LoRa transmission is considered correctly detected and classified if it has the same SF as specified in the ground truth and if its central frequency deviates by no more than 50 kHz from the specified ground truth. Only correctly detected and classified LoRa transmissions are included when calculating MACFD and MABD.

(1) Implementation of LoRa IoT nodes and transmission orchestrator

**Figure 10:**
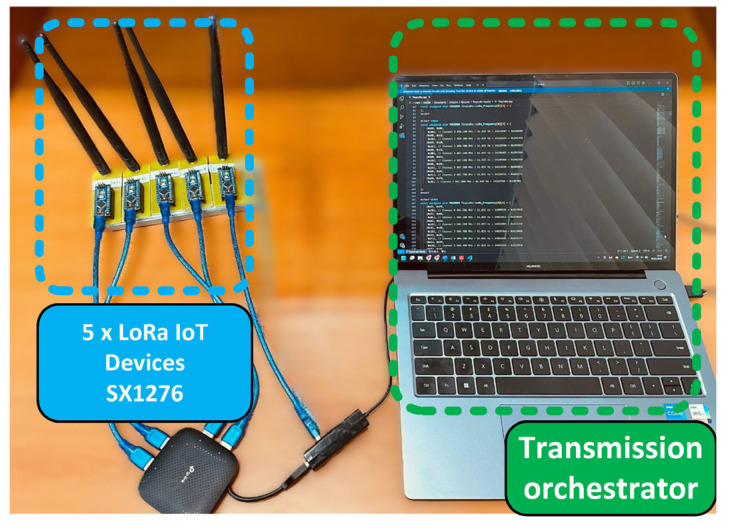
Transmission orchestrator deployment for live evaluation of SS framework.

The transmitter part of the test setup consists of five IoT nodes based on LoRa transceivers. Each node includes an Atmega328p microcontroller [39] connected to an SX1276 LoRa transceiver [40] through the SPI interface. The transceivers use omnidirectional antennas with a 0.5 dB gain, which artificially increases the effective distance between the transmitter and receiver. Custom firmware on each microcontroller enables control of the communication channel and SF via the serial interface. Figure 10 shows the transmission orchestrator deployment setup.

For the LoRa packet payload, a random byte sequence of variable length (ranging from 1 to 51 bytes) is used, ensuring a broad distribution of transmitted LoRa symbols. There is no delay between consecutive LoRa transmissions; once configured by the transmission orchestrator, a node continuously transmits LoRa packets with random payloads using the assigned SF and channel until instructed otherwise. This approach ensures a controlled environment for assessing the detection performance of the SS framework, eliminating the need for signal capture synchronization between the transmitting side and the receiving side within a transmission frame.

The transmission orchestrator is connected to the five IoT LoRa nodes via USB-to-serial converters. At the transmission orchestrator level, a list of LoRa channels and SFs is defined. In this test setup, LoRa channels span from 867.7 MHz to 868.5 MHz, specifically CH6, CH7, CH0, CH1, and CH2, with central frequencies of 867.5 MHz, 867.9 MHz, 868.1 MHz, 868.3 MHz, and 868.5 MHz, respectively, and a bandwidth of 125 kHz. Each IoT node is assigned a unique channel and SF, forming a transmission frame. This configuration is recorded in a ground truth file for later comparison with detection results.

After the IoT nodes are configured, the transmission orchestrator signals the receiver via an IP connection that the SDR can begin capturing radio signal samples. The SDR forwards the recorded IQ samples and forwards them to the SS framework, which detects, classifies, and generates channel analytics for all LoRa transmissions within the current frame. Once the receiver confirms successful capture, the transmission orchestrator randomly reassigns LoRa channels and SFs to the IoT nodes, and the cycle repeats. This setup ensures a controlled environment for accurate performance evaluation of the SS framework.

(2) Implementation of SS framework for LoRa modulation detection

In this test setup, the receiver component consists of an Ettus USRP N310 SDR, connected via Ethernet to an Alienware Aurora R10 host machine (AMD Ryzen 9 3950X, 64GB DDR4, 2 x GeForce RTX 3060 6GB). The SDR is configured to a center frequency of 868.1 MHz and a sampling rate of 1 MS/s, ensuring coverage of the LoRa channels defined at the transmission orchestrator. The SDR uses a VERT900 antenna [41] with a gain of 3 dB.

On the host machine, the SS framework, encompassing the SDR interface, TDCA, CNN, and channel analytics algorithms, runs in a MATLAB [42] environment. Once the framework receives confirmation that the IoT nodes have been successfully configured, the SDR captures 32,768 IQ samples (32.76 ms of radio signal at a 1 MS/s sample rate) in burst mode to prevent data overflows or underflows. The captured signal is processed by the SS framework, and the detection results, which include the identified SF, central frequency, and bandwidth, are recorded in a file for performance evaluation against the ground truth. After processing the current transmission frame, the SS framework signals the transmission orchestrator via IP to update the IoT nodes configuration, repeating the cycle.

(3) Test setup methodology and performance metrics computation

As presented in the transmitter and receiver description, the test setup was designed to provide a controlled environment for accurately assessing the performance of the SS framework in a real-world operation context. To achieve this, LoRa transmission frames with known parameters were created, and both the transmitter and receiver were synchronized via an IP connection to ensure that the signal captured at the receiver matches the current configuration set by the transmission orchestrator.

In this test setup, 100 LoRa transmission frames were generated and processed by the SS framework, with each frame containing five LoRa transmissions on different channels and using different spreading factors. During each iteration, the transmission orchestrator first assigns a unique channel and SF to the IoT nodes, storing this information in a ground truth file. The receiver is then signaled to capture the signal and pass it to the SS framework, which records the detection results and channel metrics in a detection results file. After processing the current frame, the SS framework signals to the transmission orchestrator that it can configure the next frame. To simplify the performance metric calculations, a “new frame” separator is inserted in both the ground truth file and the detection results file, allowing clear demarcation of individual transmission frames.

After obtaining the ground truth and detection results file, the performance metrics are computed according to the following criteria:

- A radio transmission is considered correctly detected if its central frequency deviates by no more than 50 kHz from the specified ground truth. If the deviation exceeds 50 kHz or it is not detected and clustered by the TDCA, the transmission is labeled as undetected.
- A LoRa transmission is considered correctly detected and classified if its attributed label matches the ground truth label and its central frequency deviates by no more than 50 kHz from the ground truth.
- For the MACFD and MABD metrics, only LoRa transmissions that are both correctly detected and correctly classified are included.

From a total of 500 LoRa transmissions sent by the transmission orchestrator, the SS framework successfully detected 481, resulting in a detection accuracy of 96.2%. Upon closer inspection, most of the undetected transmissions occurred on LoRa CH6 and LoRa CH2, which are located at the edges of the analyzed frequency band. This issue may arise from the frequency response of the analog and digital filters in the SDR’s RX path. In practical scenarios, it can be mitigated by sampling a wider frequency band than the specific band of interest.

**Figure 11:**
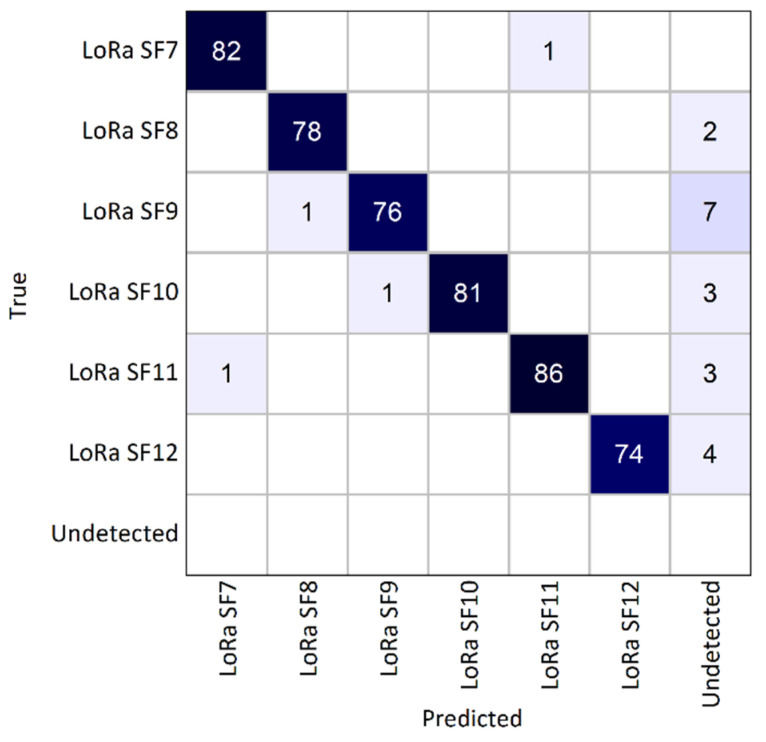
Confusion matrix for real operating environment (test scenario C).

The SS framework achieved a precision of 99.16%, aligning with the results obtained from the empirically generated dataset and indicating robust performance of the developed CNN in real-world inference scenarios. The obtained recall value was 95.4%. In the confusion matrix shown in Figure 11, where compared to previously presented confusion matrices, an “undetected” class was added, and we can see that there were only a few misclassifications (one SF7 as SF11, one SF9 as SF8, one SF10 as SF9, and one SF11 as SF7). Most of the undetected transmissions involved SF9 and SF12. Nevertheless, the well-defined diagonal in the confusion matrix confirms the framework’s high level of accuracy in LoRa detection and classification.

When computing the MACFD metric, the mean deviation in the central frequency was 9.13 kHz, which is 7.3% of a LoRa transmission’s total bandwidth. The MABD metric showed a mean bandwidth deviation of 10.2 kHz, corresponding to 8.16% of the total bandwidth.

## 7. Conclusions

This paper proposes and evaluates a novel hybrid SS framework for LoRa SF detection and communication channel analytics computation. By combining traditional signal processing methods with AI techniques, the developed framework is capable of detecting and classifying multiple simultaneous LoRa transmissions in a wideband signal based solely on the input IQ samples. The live inference evaluation demonstrated a high level of performance, achieving a detection accuracy of 96.2%, a precision of 99.16%, and a recall of 95.4%. In addition, the low mean frequency deviation (MACFD of 9.13 kHz) and reduced communication channel bandwidth deviation (MABD of 10.2 kHz) highlight the performance of the proposed SS framework.

The developed SS framework aims to address the increasing challenges of the ever-growing large-scale, high-density IoT wireless sensor networks based on LoRa communication in terms of packet collision, interference, and reduced channel capacity within the constrained spectral resources. The ability to dynamically allocate communication channels and accurately analyze channel conditions ensures the optimal performance, scalability, and reliability of IoT networks. In addition, the framework’s flexible architecture, achieved by separating the LoRa SF detection processing pipeline from the communication channel analytics processing pipeline, allows it to adapt to being used for various communication standards beyond LoRa, enhancing its applicability in diverse IoT scenarios.

However, the framework revealed certain limitations, primarily related to the TDCA algorithm, which relies on classical energy detection and rule-based clustering methods. This can lead to missed transmissions, especially under noisy or difficult conditions. Future research should explore replacing TDCA with a more advanced 1D semantic segmentation neural network capable of learning and extracting radio transmissions based on clustering patterns even under challenging conditions.

Another limitation is the currently fixed 1 MHz sampling rate required for the CNN input. To address this, future iterations of the SS framework will include training datasets with varying sampling rates, thereby enhancing the ability of the CNN to process inputs directly without explicit resampling operations. Addressing these issues will significantly improve the framework’s versatility, accuracy, and applicability in dynamic IoT environments, ensuring the continued growth and robustness of LoRa-based IoT networks.

## Data Availability

The original data presented in the study are openly available in spectrumHiveDatasets at https://github.com/WirelessLabUSV/spectrumHiveDatasets/blob/main/spectrumHive_LoRa.md or [33] reference of the paper.
